# LITAF Mutations Associated with Charcot-Marie-Tooth Disease 1C Show Mislocalization from the Late Endosome/Lysosome to the Mitochondria

**DOI:** 10.1371/journal.pone.0103454

**Published:** 2014-07-24

**Authors:** Andressa Ferreira Lacerda, Emily Hartjes, Craig R. Brunetti

**Affiliations:** Biology Department, Trent University, Peterborough, Ontario, Canada; Centro de Investigación Príncipe Felipe – CIPF, Spain

## Abstract

Charcot-Marie-Tooth (CMT) disease is one of the most common heritable neuromuscular disorders, affecting 1 in every 2500 people. Mutations in LITAF have been shown to be causative for CMT type 1C disease. In this paper we explore the subcellular localization of wild type LITAF and mutant forms of LITAF known to cause CMT1C (T49M, A111G, G112S, T115N, W116G, L122V and P135T). The results show that LITAF mutants A111G, G112S, W116G, and T115N mislocalize from the late endosome/lysosome to the mitochondria while the mutants T49M, L122V, and P135T show partial mislocalization with a portion of the total protein present in the late endosome/lysosome and the remainder of the protein localized to the mitochondria. This suggests that different mutants of LITAF will produce differing severity of disease. We also explored the effect of the presence of mutant LITAF on wild-type LITAF localization. We showed that in cells heterozygous for LITAF, CMT1C mutants T49M and G112S are dominant since wild-type LITAF localized to the mitochondria when co-transfected with a LITAF mutant. Finally, we demonstrated how LITAF transits to the endosome and mitochondria compartments of the cell. Using Brefeldin A to block ER to Golgi transport we demonstrated that wild type LITAF traffics through the secretory pathway to the late endosome/lysosome while the LITAF mutants transit to the mitochondria independent of the secretory pathway. In addition, we demonstrated that the C-terminus of LITAF is necessary and sufficient for targeting of wild-type LITAF to the late endosome/lysosome and the mutants to the mitochondria. Together these data provide insight into how mutations in LITAF cause CMT1C disease.

## Introduction

Lipopolysaccharide-induced tumor necrosis factor-alpha factor (LITAF), also known as SIMPLE (small integral membrane protein of the lysosome/late endosome) is a 161 amino acid protein that is composed of two very distinct termini [1]–[3]. The N-terminus of LITAF contains two PPXY domains (where X is any amino acid) responsible for binding to WWOX, NEDD4, TSG101, STAM1, Hrs and Itch [4]–[8]. The C-terminus of LITAF is 68 amino acids long and contains a modified RING-domain including a CX2C domain, a hydrophobic region (approximately 25 amino acids long) and a HXCX2C motif [1]. This interrupted RING-finger domain has been termed the SIMPLE-like domain (SLD) [1].

LITAF has been implicated in Charcot-Marie-Tooth (CMT) disease, which is one of the most common heritable neuromuscular disorders, affecting approximately 1 in 2500 people. The demyelinating type, CMT1, is divided into several subgroups (A–E), depending on the specific gene influencing the progression of the disease. CMT1A (70%–80%) involves duplication of PMP22 [9], CMT1B (6%–10%) is associated with point mutations in myelin protein zero (MPZ), CMT1C (1%–2%) is associated with mutations in LITAF, and CMT1D (<2%) is associated with mutations in EGR2. Finally, CMT1E (<5%) is associated with point mutations in PMP22 while CMT2E/1F (<5%) is associated with mutations in neurofilament light polypeptide (NEFL) [10], [11]. LITAF mutations associated with CMT occur mostly in the C-terminus of LITAF (SLD), specifically around the hydrophobic domain that is flanked by the two CX2C motifs that compose the consensus sequence of the SLD. The clustering of mutations within the conserved SLD of LITAF suggests a functional significance for this area of LITAF, however, the mechanism involved in how LITAF causes CMT subtype 1C is unknown.

Recent studies [12] have demonstrated that LITAF is necessary for recruitment of ESCRT components to endosomal membranes and for regulating endosomal trafficking and signaling attenuation of ErbB receptors. In addition, LITAF has been shown to regulate the production of exosomes and mutations in LITAF alter exosome production [13]. Improper formation of MVB and accumulation of lysosomes, in part, contribute to the reduced production of exosomes [13]. It was suggested that LITAF's ability to regulate ErbB trafficking and signaling is inhibited by LITAF mutants associated with CMT1C through a loss-of-function dominant negative mechanism, resulting in longer activation time of ERK1/2 signaling [12]. Further, Lee et al [12] suggest that LITAF mutants retain the ability to bind STAM1, Hrs and TSG101, which play a vital role in the formation of multivesicular bodies (MVB) by binding and clustering ubiquinated proteins and/or receptors on the surface of the cell.

**Figure 1:**
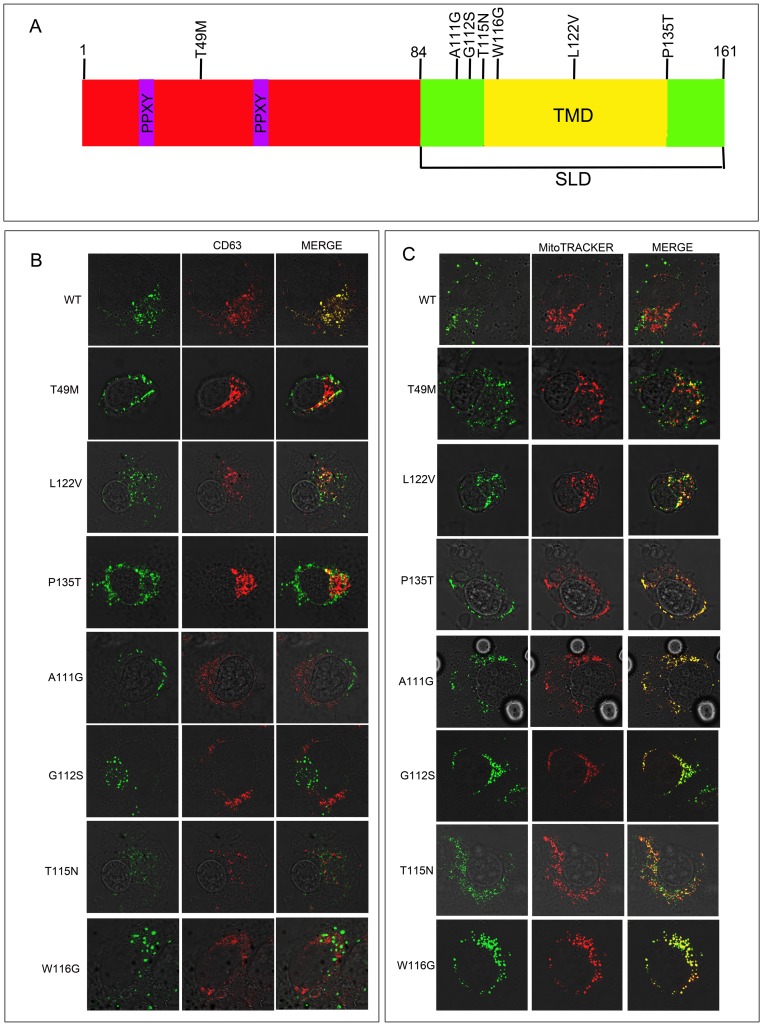
LITAF mutations associated with CMT disease. (A) The position of mutations in LITAF that cause CMT1C are shown above the protein. The PPXY domains are shown in purple and the transmembrane domain (TMD) of the simple like domain (SLD) is shown in yellow. BGMK cells were transiently transfected with myc-tagged WT LITAF, T49M, A111G, G112S, T115N W116G, L122V or P135T. (B) Twenty-four hours post-transfection the cells were visualized using indirect immunofluorescence to detect LITAF using anti-myc antibodies (green) and the late endosome/lysosome was visualized using anti-CD63 (red) or (C) Twenty-four hours post-transfection the cells were incubated with MitoTRACKER Red FM for 2hrs. The cells were then fixed and permeabilized indirect immunofluorescence was performed to detect LITAF using anti-myc antibodies (green) and the mitochondria was visualized using MitoTRACKER Red FM. (red).

Genetic studies have identified 9 mutations of LITAF related to CMT1C (T49M, I92V, A111G, G112S, T115N, W116G, L122V, P135S and P135T) [11], [14]–[17]. It has been noted [8] that 7 of the 9 mutations (A111G, G112S, T115N, W116G, L122V, P135S and P135T) associated with CMT1C are present in or around a potential LITAF transmembrane domain (TMD) (Figure 1A) in the C-terminal SLD domain, and it was shown that LITAF is a membrane protein that requires the C-terminus for this membrane association. The lack of ER-targeting signal sequence, and the location of the transmembrane domain suggest that LITAF is likely to undergo post-translational insertion as a C-terminal-tail-anchored membrane protein [8], [18], [19]. Lee et al., [8] have demonstrated that two mutants of LITAF, W116G and P135T, that cause CMT1C result in mislocalization of LITAF from the endosome to the cytosol. This would suggest that these mutants of LITAF are not present in the appropriate location in the cell resulting in impairment of endosomal trafficking and sorting which could be responsible for the CMT1C disease phenotype [12].

In this study we explored the localization of LITAF mutants T49M, A11G, T115N G112S, W116G, L122V and P135T. We undertook experiments to understand not only their intracellular localization but also potential targeting pathways for LITAF and its mutants. Finally, we investigated the effect of cells heterozygous for wild-type and mutant LITAF to determine whether the disease phenotype would be produced in these cells.

## Materials and Methods

### Reagents, cell lines, and antibodies

Baby green monkey kidney (BGMK) cells were obtained from the American Type Culture Collection (ATCC; Manassas, VA) and were maintained at 37°C with 5% CO2 in Dulbecco's modified Eagle's medium (DMEM; HyClone, Ottawa, ON) supplemented with 7% FBS, 2 mM L-glutamine, penicillin (100 U/mL), and streptomycin (100 µg/mL). LAN-5 cells (neuronal cell line) were a gift from Dr. David Kaplan (Sick Kids Hospital, Toronto, ON) and were maintained at 37°C with 5% CO2 in Roswell Park Memorial Institute 1640 medium (RPMI, Life Technologies, Burlington, ON) supplemented with 10% FBS and penicillin (100 U/mL), and streptomycin (100 µg/mL). Cells used for immunofluorescence were transfected using a polyethylenimine (PEI) reagent using 5 µg plasmid/10 cm^2^ plate and a PEI:DNA ratio of 4∶1. The following antibodies/probes were used during immunofluorescence: 9E10 myc monoclonal antibody obtained from Roche (dilution - 1/100; Indianapolis, IN); monoclonal antibody against FLAG (M2) from Sigma (dilution - 1/500; Oakville, ON); anti-EGF from Santa Cruz Biotechnology (dilution – 1/50; Santa Cruz, CA); FITC/Cy3/Cy5-conjugated goat anti-mouse or anti-rabbit immunoglobulin G (IgG) from Jackson ImmunoResearch Inc. (dilutions – 1/100, 1/200, 1/100 respectively; West Grove, PA); anti-CD63 antibody from Invitrogen (dilution – 1/100; Burlington, ON). LysoTRACKER DND-99 (Molecular Probes, Burlington, ON). MitoTRACKER Red FM (Molecular Probes, Burlington, ON) which selectively concentrates in active mitochondria and retained during cell fixation, providing an advantage over conventional fluorescent stains for mitochondria, such as tetramethylrosamine and rhodamine 123, which are readily sequestered by functioning mitochondria, but easily washed out of cells once the mitochondria experience a loss in membrane potential. Both LysoTRACKER DND-99 and MitoTRACKER Red FM were prepared according to manufactures recommendations. Stock solutions were prepared by dissolving the reagents in DMSO to a final concentration of 1 mM. The working concentrations were obtained by dissolving the stock in fresh cell culture media to a final concentration of 200 nM.

### LITAF mutants

Human LITAF WT (NP001129945.1) and LITAF mutants associated with CMT1C where synthesized by GeneScript (T49M, A111G, G112S, T115N, W116G, L122V and P135T; Piscataway, NJ). A myc-tag was added to the N-terminus of the protein to facilitate imaging.

Synthesis of the LITAF SLD constructs - LITAF WT and G112S mutant were amplified using a 50 µL PCR mixture containing 1X PCR buffer (Invitrogen), 3.0 mM MgCl2 (Invitrogen), 0.2 mM forward and reverse primers, and 2.5 U Taq DNA polymerase (5 U/µL; Invitrogen). LITAF WT and mutations were used as template DNA for the following primers: 5′- ATGGAGCAGAAACTGATTAGTGAAGAAGACCTGCCCATCACTTTCCTGGATCGGCCCATT -3′ (LITAF-forward), 5′-TACCAGTCT TTGTAAGTTCC -3′ (LITAF-reverse). The cycling conditions used were: 94°C for 30 seconds, 52°C for 30 seconds, 72°C for 90 seconds for 30 cycles. The resulting PCR product was cloned into pTARGET (Promega, Madison, WI).

### Sequencing

In order to confirm that transformation into cloning vectors was performed correctly and to identify potential LITAF mutations, LITAF samples were prepared, and sent for sequencing at the Robarts Institute (London, ON). Once sequencing was completed, data was analyzed using Codon Aligner 4.0.4 (Centerville, MA)

### Transfections

BGMK or LAN-5 cells were grown to 70% confluency and transfected using a polyethylenimine (PEI) reagent. Five micrograms of LITAF DNA (WT or mutant) was diluted with 200 µl of serum free DMEM. PEI was added in a 4∶1 ratio of PEI:DNA and left to incubate at room temperature for 15 minutes. Transfection mix was then added to cells along with fresh DMEM.

### Exposure to Brefeldin A

Wild type or mutant LITAF were transfected into cells, and 4 hours post transfection, the cells were treated with 10 mM brefeldin A (BFA). First BFA was left on the cells for 24 hours, probed for mitochondria (MitoTRACKER Red FM) and fixed for immunofluorescence. The cells were then fixed, and indirect immunofluorescence was performed to visualize LITAF (WT or mutant) and endoplasmic reticulum to determine the subcellular localization of LITAF and mutants in the presence and absence of BFA.

### Immunofluorescence

Cells were fixed using 3.7% paraformaldehyde solution in phosphate buffer solution (PBS) and permeabilized using a 0.1% Triton X-100 solution in PBS. Cells were blocked for 2 hours at room temperature in block buffer (5% bovine serum albumin (BSA) (w/v), 50 mM Tris HCl (pH 7.4), 150 mM NaCl, 0.5% NP-40 (v/v)) followed by several 5-minute washes in wash buffer (1% bovine serum albumin (BSA) (w/v), 50 mM Tris HCl (pH 7.4), 150 mM NaCl, 0.5% NP- 40 (v/v)). Primary antibody diluted in wash buffer was incubated on cells for 1 hour at room temperature. The primary antibody was removed following several 5-minute washes in wash buffer and secondary antibody was diluted in wash buffer and applied to cells. The secondary antibody was left on cells for 1 hour at room temperature in darkness before removal with several 5-minute washes in wash buffer. Cells were mounted using VECTASHIELD (Vector Labs, Burlington, ON) and fluorescence was detected using a Leica DM SP2 confocal microscope (Leica, Wetzlar, Germany). Images were assembled using Adobe Photoshop CS4 (Adobe, San Jose, CA). Experiments were performed at last 3 times to confirm observations and representative images are shown.

## Results

### CMT1C LITAF mutations mislocalize to the mitochondria

**Figure 2:**
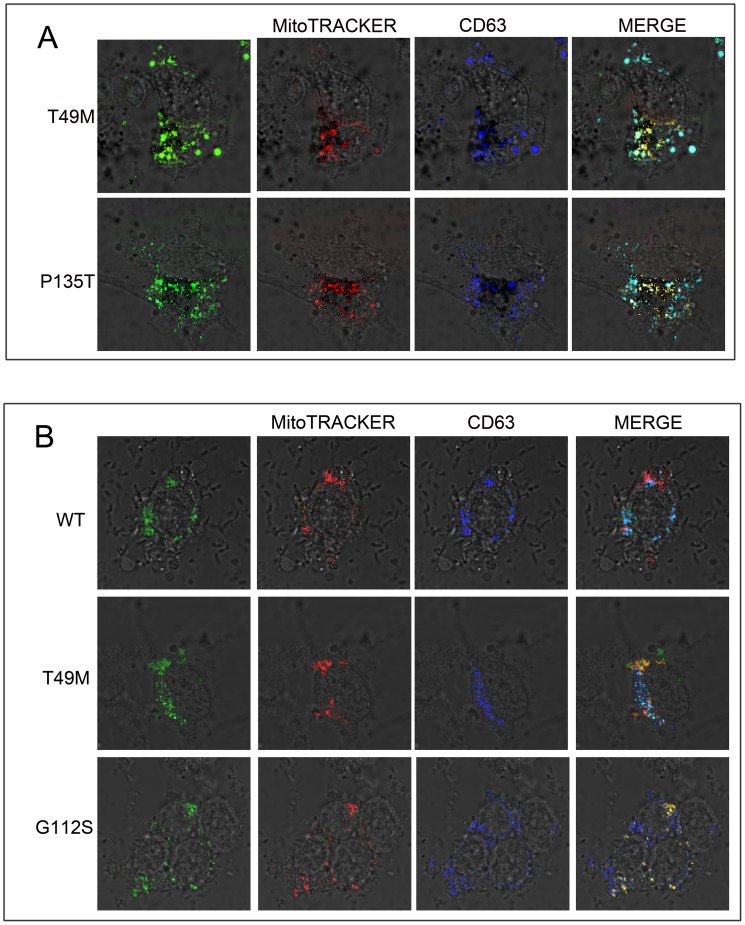
LITAF mutants show different localization then WT LITAF. (A) BGMK cells or (B) LAN-5 cells were transfected with myc-tagged WT LITAF, T49M, G112S, or P135T. Twenty- four hours after transfection, the cells were fixed and stained with anti-myc-antibodies (green), mitochondria was visualized using MitoTRACKER Red FM (red), and late endosome/lysosomes were stained using CD63 (blue). Nuclei were visualized using differential interface contrast (DIC).

We were interested in determining the subcellular localization of wild type (WT) LITAF and the LITAF mutants: T49M, A111G, G112S, T115N, W116G, L122V and P135T to better understand how LITAF causes CMT1C disease. We transfected BGMK cells with WT LITAF or the T49M, A111G, G112S, T115N, W116G, L122V or P135T mutants. We screened the LITAF constucts using markers to each of the organelles in the cell. Consistent with other reports [7], [20] WT LITAF co-localized with the late endosome/lysosomal marker CD63 (Figure 1B), suggesting that WT LITAF is normally present in the late endosome/lysosomes. In contrast, the LITAF mutants A111G, G112S, T115N and W116G localize entirely with the mitochondrial marker MitoTRACKER Red FM (Figure 1C) and not the late endosome/lysosome marker CD63 (Figure 1B). On the other hand, LITAF mutants T49M, L122V and P135T partially co- localized with both the late endosome/lysosomal (Figure 1B) and mitochondrial marker (Figure 1C). Triple labeling experimets (LITAF, CD63 (late endosome/lysosome), and MitoTracker) showed partial mislocalization with a portion of the total protein present in the late endosome/lysosome and the remainder of the protein localized to the mitochondria (Figure 2A). We showed that both wild-type and mutant LITAF show similar stability in cells via Western Blot (data not shown). Since CMT1C results in defects in neuronal cells [14], we examined the localization of LITAF and its mutants in LAN-5 (a neuroblasmtoma cell lines) cells, which are neuronal cells. In LAN-5 cells T49M and G112S mutants behave similarly to BGMK cells with the T49M mutant showing partial mislocation and G112S fully mislocalizing to the mitochondria (Figure 2B).

### LITAF mutants are dominant

**Figure 3:**
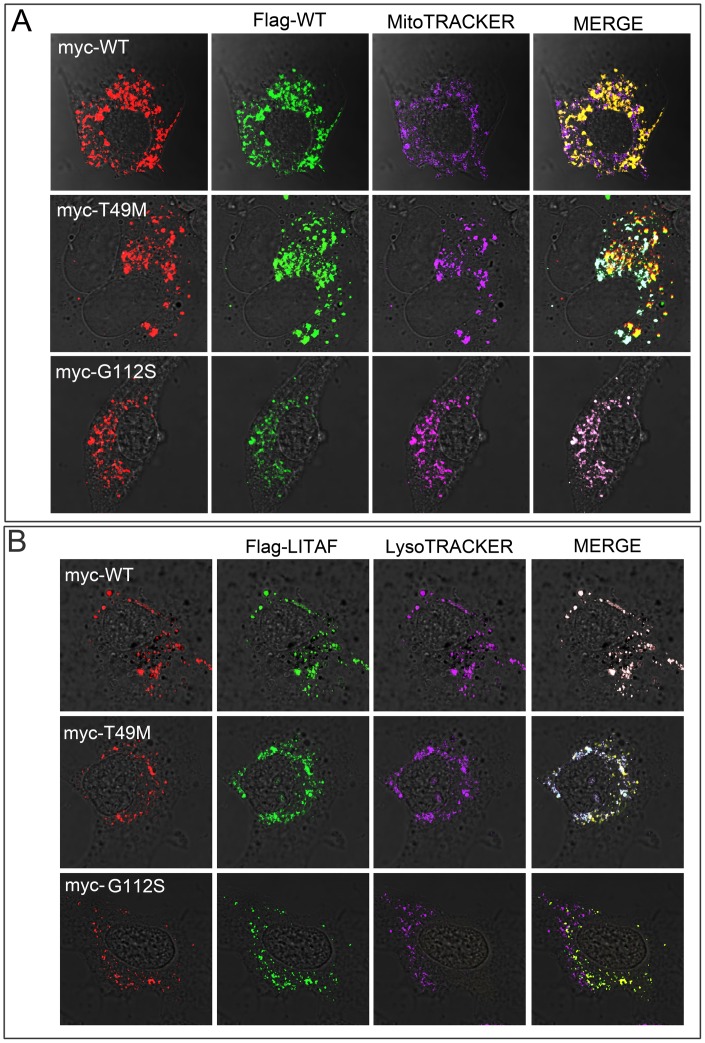
CMT1C LITAF mutations act in a dominant negative fashion. BGMK cells were co-transfected with flag-tagged WT LITAF and either myc-tag WT LITAF, T49M and G112S. Twenty- four hours after transfection, the cells were probed with MitoTRACKER Red FM or LysoTRACKER DND-99 for 2 hrs then fixed and stained with anti-flag-antibodies (green), anti-myc-antibodies (red) and (A) the mitochondria was visualized using MitoTRACKER Red FM (purple) or (B) the late endosome/lysosomes were visualized using LysoTRACKER DND-99 (purple).

For the remainder of the analysis, we focused on 2 representative mutants of each misolocation category – T49M represents a mutant that partially mislocalizes and G112S represents a mutant the completely mislocalizes to the mitochondria. Since WT LITAF localizes to the late endosome/lysosome and the LITAF mutants were found to be associated with the mitochondria, we wondered whether the LITAF mutants could act in a dominant negative fashion to cause mislocalization of WT LITAF. BGMK cells were co-transfected with flag-tagged WT LITAF and either myc-tagged WT LITAF, T49M or G112S. Twenty-four hours post transfection, the cells were fixed and stained and the subcellular localization of LITAF was determined. We found that WT LITAF co-localized with LITAF mutants when co-transfected (Figure 3). We observed that when WT LITAF and T49M were transfected into the cells, WT LITAF was localized to the mitochondria (Fig 3A) and the late endosome/lysosome (Figure 3B) consistent with the localization of the T49M mutant alone (Figure 2). Similar results were obtained with P135T (data not shown). When WT LITAF and G112S were co-transfected, WT LITAF completely mislocalized from the late endosome/lysosome to the mitochondria (Figure 3). Again, consistent with the localization of G112S alone (Figure 1C). These data suggest that heterozygous individuals containing a mutant and WT copy of LITAF will exhibit a CMT1C phenotype as the wild-type copy is mislocalized by the mutant copy.

### Wild-type LITAF transits though the secretory pathway

**Figure 4:**
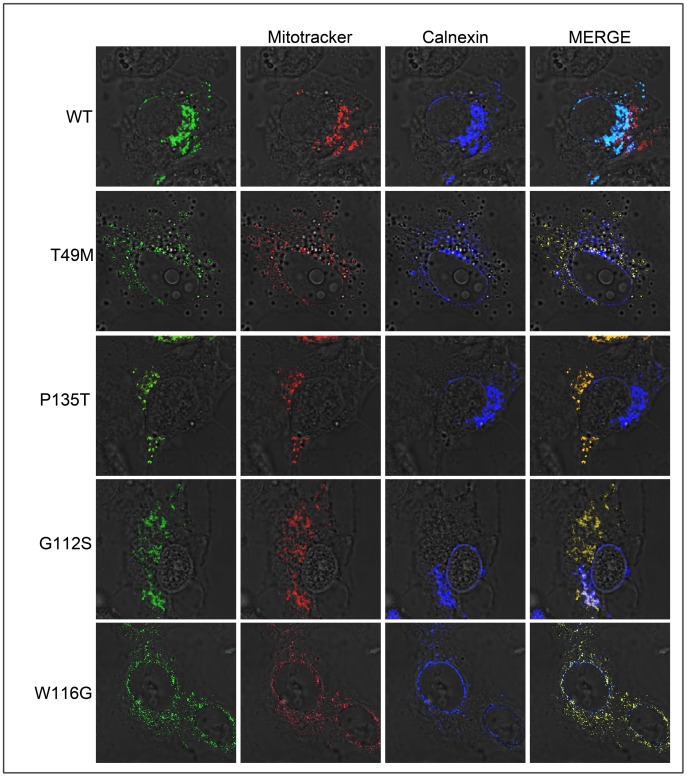
LITAF mutations do not transit though the secretory pathway. BGMK cells were transiently transfected with myc-tagged WT LITAF, T49M, G112S, W116G or P135T. Four hours post transfection the cells were exposed to 10 mM of Brefeldin A. Twenty-four hours post-transfection the cells were probed with MitoTRACKER Red FM for 2 hrs then fixed and permeabilized. Indirect immunofluorescence was performed and WT and mutant LITAF were detected using anti-myc antibody (green), mitochondria were visualized using MitoTRACKER Red FM (red) and the ER was visualized using anti-calnexin antibody (blue).

We were interested in determining the trafficking pathway of WT LITAF to the late endosome/lysosome. Brefeldin A (BFA) is a fungal metabolite demonstrated to reversibly interfere with anterograde transport from the endoplasmic reticulum to the Golgi apparatus [21]. Treatment with BFA leads to a rapid accumulation of proteins within the ER and collapse of the Golgi stacks. Therefore, proteins that transit through the secretory pathway will be trapped in the ER upon BFA treatment. If proteins are directly targeted to the late endosome/lysosome from the cytoplasm, then BFA will not affect LITAF targeting. However, if proteins travel though the secretory pathway to the late endosome/lysosome, then they will be retained in the ER upon BFA treatment. Therefore, we transfected WT or mutant LITAF into cells, and 4 hours post transfection the cells were treated with 10 mM BFA. Twenty-four hours post treatment with BFA the cells were fixed, and indirect immunofluorescence was performed to visualize the LITAF (WT or mutant), endoplasmic reticulum, and mitochondria to determine the subcellular localization of LITAF and its mutants in the presence and absence of BFA. Our results show that upon BFA treatment, WT LITAF co-localized with the ER marker (calnexin) (Figure 4) suggesting that LITAF transits through the secretory pathway to the late endosome/lysosome. In contrast, all the LITAF mutants T49M, G112S, W116G, and P135T (Figure 4) completely localized to the mitochondria upon BFA treatment, suggesting that LITAF mutants do not transit through the secretory pathway but are directly targeted to the mitochondria.

### The simple like domain (SLD) of LITAF is sufficient for directing the subcellular localization of LITAF

**Figure 5:**
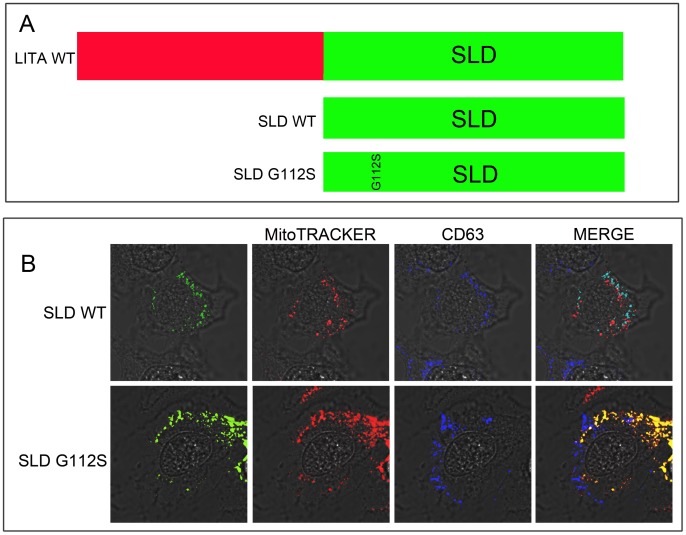
The simple-like domain (SLD) is responsible for the subcellular localization of LITAF. (A) Schematic of WT LITAF and its truncated versions SLD-WT and SLD G112S. (B) BGMK cells were transiently transfected with myc-tagged SLD-WT or SLD0-G112S. Twenty-four hours post-transfection the cells were incubated with MitoTRACKER Red FM for 2 hrs then fixed and permeabilized. The cells were stained with anti-myc-antibodies (green) anti-CD63 antibodies (blue) and the mitochondria were visualized using MitoTRACKER Red FM (red).

We were interested in determining if the SLD domain (amino acids 84–152, Figure 5A) of LITAF was necessary and sufficient for determining the subcellular localization of LITAF. BGMK cells were co-transfected with SLD-WT or SLD-G112S mutant. Twenty-four hours post transfection, the cells were fixed and stained and the subcellular localization of the proteins was determined. We observed that SLD-WT co-localized with the late/endosome/lysosomal marker (CD63) (Fig 5B) while SLD-G112S co-localizes with the mitochondrial marker (MitoTRACKER Red FM) (Fig 5B). These results are in agreement with the localization of the full-length protein. Therefore, these data suggest that the SLD is responsible for determining the subcellular localization of LITAF and a single amino acid change within the SLD is sufficient for changing LITAF's localization.

## Discussion

To gain a greater understanding of CMT1C disease, we characterized a variety of LITAF mutants associated with the disease. In this study, we observed that mutants in LITAF that cause CMT1C have an altered intracellular localization. WT LITAF localized to the late endosome/lysosome while LITAF mutants A111G, G112S, W116G, and T115N showed complete localization to the mitochondria and LITAF mutants T49M, L122V, and P135T co-localize with both late endosome/lysosome and mitochondria. Interestingly, the mutants that cause complete mislocalization to the mitochondria are clustered near the 5′end of the transmembrane domain within the SLD (Fig 1A). While mutants further away from this region cause only partial mislocalization. Previous reports (8) have suggested that LITAF mutants W116G and P135T were localized to the cytoplasm. However, the cytoplasmic staining reported is quite punctate and could be consistent with mitochondria localization [8]. Interestingly, not only are LITAF mutants targeted to the mitochondria but SGIV136, a viral protein homolog of LITAF, also shows localization to the mitochondria [22], [23] suggesting that there is a preferential mislocalization of LITAF specifically to the mitochondria. Other studies [8], [12] described that LITAF mutants promote the formation of LITAF-positive aggresomes. We have previously investigated LITAF in BGMK cells and demonstrated that endogenous LITAF co-localizes with aggresomes [20]. However, in the present study, we did not observe localization of the LITAF mutants in any other subcellular location other than late endosome/lysosomes and mitochondria.

Our report is the first comprehensive analysis of LITAF mutants. All the mutants we analyzed (T49M, A111G, G112S, T115N, W116G, L122V and P135T) showed at least partial mislocalization to the mitochondria. Interestingly, none of the mutants showed wild-type distribution in the cell suggesting that CMT1C is caused by mislocalization of LITAF from its site of action [1], [4], [6], [24]. Since LITAF's proposed function is to recruit ESCRT components to endosomal membranes and regulate endosomal trafficking and decrease of ErbB receptors [12], mutations that alter the localization of LITAF could result in the inhibition of endosomal trafficking or function, which could impair lysosomal degradation of proteins resulting in CMT1C. For example, loss of function of LITAF results in impairment ErbB trafficking and signaling [12]. It has also been suggested that LITAF plays an important role in the formation of multivesicular bodies (MVB) which mediate endosomal trafficking [13]. Zhu et al [13] demonstrated that LITAF mutants A111G, G112S, T115N, W116G, P135T and P135S had defects in exosome secretion while mutants T49M and L122V did not show these defects. We believe our data offers an explanation on differences in exosome secretion by the various LITAF mutants. As shown in figures 2, LITAF mutations T49M and L122V show only partial mislocalization of LITAF from the late endosomes/lysosomes suggesting there maybe some residual late endosome/lysosome function. In contrast, mutants such as A111G, G112S, T115N, and W116G are completely mislocalized and would have no late endosome/lysosomal function. Since T49, L122V, and P135T partially co-localize to their proper intracellular localization (late endosome/lysosomes), it would be interesting to explore whether these mutants produce a less aggressive form of CMT1C disease compared to the mutants that show complete mislocalization from the late ensodosome/lysosomes. Thus far, there have been no published studies that have looked into differing clinical outcomes for the different LITAF mutants related to CMT1C. Therefore, it is possible that different CMT1C mutants present different disease phenotype in a clinical setting. It should also be noted that we can't discount the possibility that LITAF mutants maybe mediating their phenotype through disruption of mitochondrial function in addition to impairment of endosome/lysosome function.

In addition to exploring how mutations in LITAF cause disease, we also demonstrated that LITAF mutants act in a dominant negative fashion to cause mislocalization of WT LITAF. Our data is consistent with the biochemical findings of Lee et al., (2012) [12] who demonstrated that the phenotype of LITAF heterozygous cells is similar to the phenotype of cells depleted for LITAF. Our data, that mutant forms of LITAF cause mislocalization of wilt-type LITAF, provides a mechanism to explain how LITAF mutants produce the dominant-negative phenotype.

**Figure 6:**
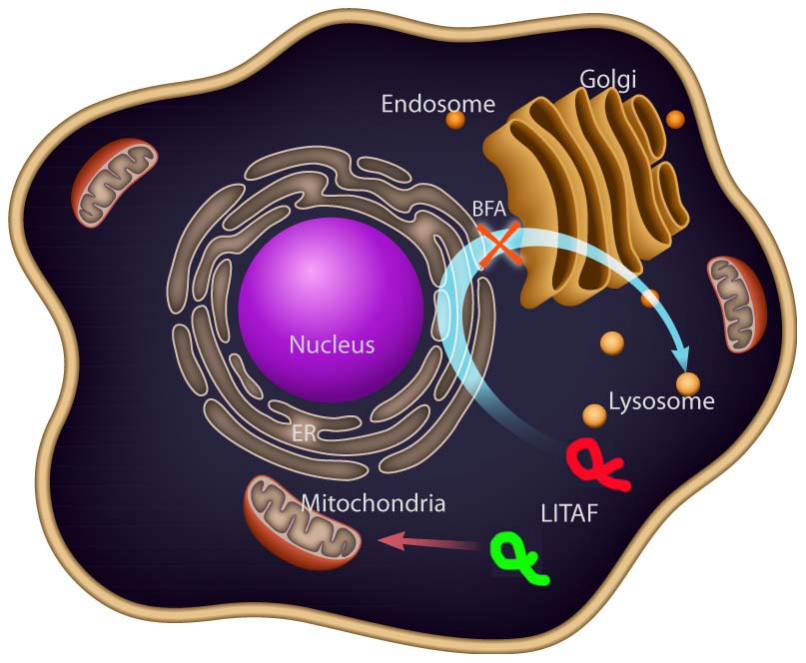
Trafficking pathways for WT LITAF and LITAF mutants. Brefeldin A experiments suggested that WT LITAF (green) transits though the secretory pathway to its final destination, the late endosome/lysosome. LITAF mutants (red) are directly targeted to the mitochondria from the cytoplasm.

One of the intriguing observations to emerge from our work was that a protein that is normally targeted to the late endosome/lysosome could be mistargeted to the mitochondria. The mitochondria and late endosome/lysosome are not normally part of the same intracellular targeting pathway and so we wondered how LITAF was targeted to these two very different organelles. We demonstrated that only WT LITAF transits through the secretory pathway using BFA treatment while LITAF mutants targeted to the mitochondria were unaffected by BFA treatment suggesting an ER-independent targeting. LITAF is suggested to be a post-translationally inserted C-tail-anchored membrane protein rather than a co-translationally inserted transmembrane protein (Fig 6) [25]. Although the molecular mechanisms underlying the post-translational insertion of C-tail-anchored membrane proteins remain poorly understood, clearly the C-terminal transmembrane domain constitutes the only membrane-targeting sequence [24] and this is supported by our own observation that the C-terminus, the SLD, is necessary and sufficient to mediate targeting of LITAF and its mutants. It is now generally accepted that after release from free ribosomes, newly synthesized tail-anchored proteins undergo rapid direct insertion into mitochondrial, peroxisomal, or ER membranes. There is a general agreement that tail-anchored protein targeting to the mitochondrial outer membrane relies on a transmembrane domain of moderate hydrophobicity flanked by positively charged residues [19], [26], [27]. Mutagenesis studies have demonstrated that both these features are essential for correct targeting, and altering either one of them causes rerouting of selected mitochondrial outer membrane tail-anchored proteins to the ER [19], [26], [27]. Nonetheless, some ER-targeted tail-anchored proteins are indistinguishable from mitochondrial outer membrane-directed ones in both transmembrane, hydrophobicity and flanking charge. Furthermore, in one case, the removal of C-terminal basic residues from an ER-targeted tail-anchored protein conferred to it the ability to bind to mitochondria in vitro. We speculate that in WT LITAF this pattern is conserved in the transmembrane domain as well, this may explain how mutations that disrupt targeting of LITAF to the secretory pathway result in LITAF being targeted to the mitochondria. The lipids of ER membranes and of mitochondria outer membrane are equally tolerant of the insertion of tail-anchored proteins with moderately hydrophobic transmembrane domains. So that mitochondria outer membrane-directed and ER directed transmembrane proteins could potentially slowly integrate into both organelles. An important problem is the mechanism through which these proteins discriminate between the mitochondrial outer membrane and the endoplasmic reticulum membrane, because the latter is the target for many polypeptides with similar topology. It has been reported that, within the tail region, charged residues downstream of the transmembrane domain play a crucial role, with basic residues favoring the targeting to the mitochondria outer membrane [24]. From mutants used in this study, all amino acid substitutions result in a change in the overall charge. For example, in the mutant T49M a tyrosine, which is hydrophilic, is replaced by a methionine, which is hydrophobic. The P135T mutant also shows a change in hydrophobicity. In the mutant G112S, a glycine has a small hydrogen substituent as its side-chain that can fit into both hydrophilic or hydrophobic environments until it is replaced by a serine, which is classified as a polar amino acid due its hydroxyl group. The W116G mutant shows a substitution of an aromatic tryptophan to a glycine.

Targeting of proteins to intracellular organelles occurs by a number of mechanisms. Proteins destined for the secretory pathway are co-translationally imported into the endoplasmic reticulum (ER). The proteins are then transported through the Golgi apparatus [28], [29] and then the trans-Golgi Network. Proteins destined for the lysosome are targeted from the trans-Golgi Network via a M6P-independent mechanism. These proteins have, in their cytoplasmic domains, lysosomal targeting sequences that interact with one or more adaptor complexes [30]. They are delivered from the trans-Golgi Network to endosomes presumably by the clathrin-coated vesicles and accumulate in lysosomes. Two of the most common lysosomal targeting sequences are tyrosine and di-leucine-based motifs [31]–[33]. Tyrosine based motifs include the consensus sequence YXXφ, where X is any amino acid and φ represents a bulky hydrophobic amino acid [32] Sorting occurs through an interaction between these signals and clathrin-coated vesicle adaptor proteins. Clathrin is a protein that mediates the formation of small vesicles. These vesicles are involved in sorting cargo within the membrane, trans-Golgi network, and early/late endosomes and adaptors mediate the recruitment and formation of clathrin-coated vesicles. LITAF contains a tyrosine based targeting sequence YXXφ (YKRL for LITAF) [34], [35], which may explain the targeting of LITAF in the secretory pathway to the late endosome/lysosome.
